# A New Contact Killing Toxin Permeabilizes Cells and Belongs to a Broadly Distributed Protein Family

**DOI:** 10.1128/mSphere.00318-21

**Published:** 2021-07-21

**Authors:** Cristian V. Crisan, Harshini Chandrashekar, Catherine Everly, Gabi Steinbach, Shannon E. Hill, Peter J. Yunker, Raquel R. Lieberman, Brian K. Hammer

**Affiliations:** a School of Biological Sciences, Georgia Institute of Technology, Atlanta, Georgia, USA; b Parker H. Petit Institute for Bioengineering & Bioscience, Georgia Institute of Technology, Atlanta, Georgia, USA; c Center for Microbial Dynamics and Infection, Georgia Institute of Technology, Atlanta, Georgia, USA; d Department of Bioengineering, University of Pennsylvania, Philadelphia, Pennsylvania, USA; e School of Physics, Georgia Institute of Technology, Atlanta, Georgia, USA; f School of Chemistry and Biochemistry, Georgia Institute of Technology, Atlanta, Georgia, USA; University of Iowa

**Keywords:** *Vibrio cholerae*, antimicrobial agents, secretion systems, toxins

## Abstract

Vibrio cholerae is an aquatic Gram-negative bacterium that causes severe diarrheal cholera disease when ingested by humans. To eliminate competitor cells in both the external environment and inside hosts, V. cholerae uses the type VI secretion system (T6SS). The T6SS is a macromolecular contact-dependent weapon employed by many Gram-negative bacteria to deliver cytotoxic proteins into adjacent cells. In addition to canonical T6SS gene clusters encoded by all sequenced V. cholerae isolates, strain BGT49 encodes another locus, which we named auxiliary (Aux) cluster 4. The Aux 4 cluster is located on a mobile genetic element and can be used by killer cells to eliminate both V. cholerae and Escherichia coli cells in a T6SS-dependent manner. A putative toxin encoded in the cluster, which we name TpeV (type VI permeabilizing effector *Vibrio*), shares no homology to known proteins and does not contain motifs or domains indicative of function. Ectopic expression of TpeV in the periplasm of E. coli permeabilizes cells and disrupts the membrane potential. Using confocal microscopy, we confirm that susceptible target cells become permeabilized when competed with killer cells harboring the Aux 4 cluster. We also determine that *tpiV*, the gene located immediately downstream of *tpeV*, encodes an immunity protein that neutralizes the toxicity of TpeV. Finally, we show that TpeV homologs are broadly distributed across important human, animal, and plant pathogens and are localized in proximity to other T6SS genes. Our results suggest that TpeV is a toxin that belongs to a large family of T6SS proteins.

**IMPORTANCE** Bacteria live in polymicrobial communities where competition for resources and space is essential for survival. Proteobacteria use the T6SS to eliminate neighboring cells and cause disease. However, the mechanisms by which many T6SS toxins kill or inhibit susceptible target cells are poorly understood. The sequence of the TpeV toxin that we describe here is unlike any previously described protein. We demonstrate that it has antimicrobial activity by permeabilizing cells, eliminating membrane potentials, and causing severe cytotoxicity. TpeV homologs are found near known T6SS genes in human, animal, and plant bacterial pathogens, indicating that the toxin is a representative member of a broadly distributed protein family. We propose that TpeV-like toxins contribute to the fitness of many bacteria. Finally, since antibiotic resistance is a critical global health threat, the discovery of new antimicrobial mechanisms could lead to the development of new treatments against resistant strains.

## INTRODUCTION

The type VI secretion system (T6SS) is a common contact-dependent antibacterial weapon employed by many Gram-negative species (1–3). Cells with an active T6SS (“killer cells” here) translocate toxic protein effectors into adjacent target cells. However, the outcome of T6SS-mediated aggression is influenced by the presence of immunity proteins in target cells, the external environment, and target cell stress responses (4–7). The harpoon-like proteinaceous apparatus is anchored to the membrane of killer cells by the membrane complex, which spans the inner membrane and periplasm (8–10). VasK, a component of the membrane complex, is essential for the assembly of the T6SS (8, 11). Hcp (hemolysin-coregulated protein) hexamers stack to form an inner tube that is capped at the distal end by a trimer of VgrG (valine-glycine repeat protein G) tip-forming proteins (2, 12, 13). PAAR (proline-alanine-alanine-arginine) proteins also interact with VgrGs and expand the toxin repertoire (14, 15). Furthermore, T6SS adaptor proteins (Taps) with a DUF4123 domain function as chaperones that deliver effectors to the apparatus (16–18). The T6SS uses a contraction mechanism that propels the inner tube and exports the toxic payload (19–21).

Vibrio cholerae is a wide-spread gastrointestinal pathogen that has caused seven cholera pandemics (22). The bacterium is found in polymicrobial marine ecosystems in association with copepods, fish, and insects (23–25). V. cholerae employs T6SS effectors that disrupt cell envelopes and contribute to pathogenicity in hosts (26–33). T6SS genes are distributed across a large cluster and two auxiliary clusters in all sequenced V. cholerae isolates (34, 35). In clinical strains like V52 and C6706, the large gene cluster encodes a VgrG tip-forming protein with a C-terminal peptidoglycan-degrading domain (32). Auxiliary clusters 1 and 2 encode the TseL lipase and VasX colicin-like effectors, respectively (28–31). An auxiliary cluster 3 is found in a subset of V. cholerae isolates and contains a peptidoglycan-degrading toxin (6, 36, 37).

Most clinical V. cholerae strains encode T6SS effectors with conserved activities (28, 34, 35). By contrast, many isolates obtained from sources other than patients harbor toxins with diverse predicted biochemical functions and may carry additional T6SS toxins compared to clinical isolates (34, 35, 38–40). We previously identified auxiliary 5 (Aux 5) T6SS clusters, which encode predicted phospholipase effectors (34, 40). Recently, several V. cholerae strains have been shown to possess an Aux 6 T6SS cluster with antibacterial activity (39). We and others have also reported that many isolates (but not C6706) contain an additional gene cluster with putative T6SS components, which we named Aux 4 (34, 40, 41). However, the ability of V. cholerae cells to use the Aux 4 cluster in T6SS-mediated bacterial competition has not been validated, and the role played by the putative effector in intoxicating target cells has not been examined.

Here, we demonstrate that the Aux 4 cluster can be used by V. cholerae to kill bacterial cells in a T6SS-dependent manner. We report that the toxin found within the cluster permeabilizes cells and disrupts the membrane potentials when expressed in the periplasm of Escherichia coli cells. A protein encoded by a gene immediately downstream of the effector neutralizes its toxicity and acts as a protective immunity factor. Finally, we show that homologs of the Aux 4 effector are found in diverse bacterial species, including human, animal, and plant pathogens. The potent antimicrobial activity of the protein and broad distribution of identified homologs suggest the toxins confer significant competition advantages to bacteria that harbor them.

## RESULTS

### The Aux 4 *tpeV*-*tpiV* are an active effector-immunity pair in strain BGT49.

**FIG 1:**
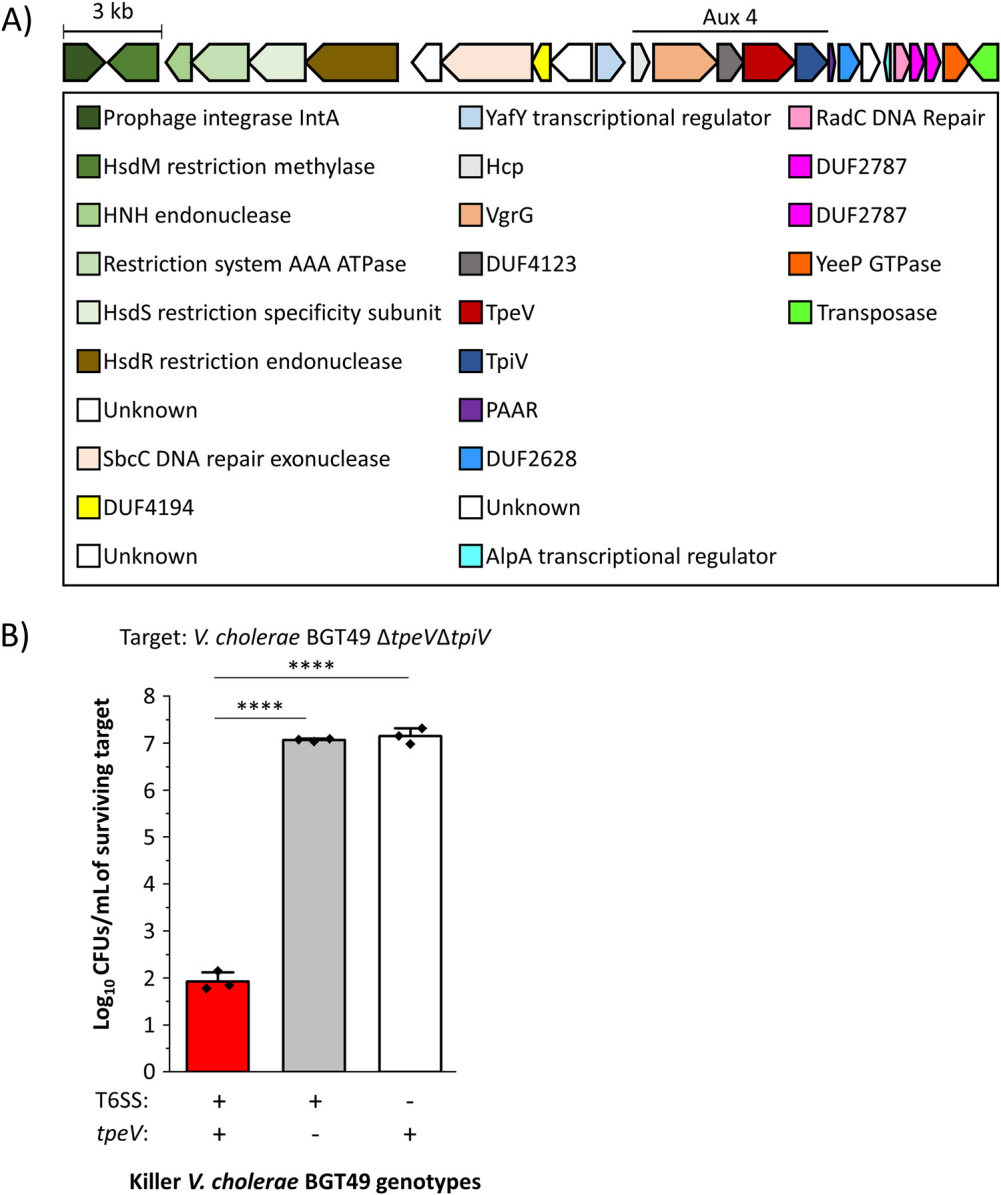
Vibrio cholerae strain BGT49 encodes the Aux 4 T6SS cluster and efficiently eliminates target bacteria in a TpeV- and T6SS-dependent manner. (A) The Aux 4 cluster encodes predicted *hcp*, *vgrG*, DUF4123-containing chaperone, effector, immunity, and *paar* genes. The cluster is found on a predicted mobile genetic element, flanked by integrase and transposase genes. (B) Target V. cholerae BGT49 Δ*tpeV* Δ*tpiV* (CC170) was cocultured with either WT, Δ*tpeV* (CC167), or Δ*vasK* T6SS^−^ (CC168) BGT49. A one-way analysis of variance (ANOVA) with a *post hoc* Tukey honestly significant difference (HSD) test was used to determine significance. ****, *P* < 0.0001.

V. cholerae strain BGT49 encodes the Aux 4 cluster in addition to the canonical T6SS large operon and auxiliary clusters 1 and 2 (Fig. 1A). The Aux 4 cluster contains the following predicted T6SS genes: an *hcp*, a *vgrG*, a DUF4123 chaperone, and a *paar* (11) (Fig. 1A). Genes coding for a putative effector toxin (which we name type VI permeabilizing effector *Vibrio* [*tpeV*]; see below) and a putative immunity protein (which we name type VI permeabilizing immunity *Vibrio* [*tpiV*]; see below) are also found within the cluster (Fig. 1A) (16, 41, 42). The Aux 4 VgrG does not contain a toxic C-terminal domain as described for the V. cholerae VgrG-1 or VgrG-3 (32, 43, 44).

Genes for a restriction modification system are found upstream of the Aux 4 cluster (Fig. 1A). Both the Aux 4 T6SS cluster and restriction modification system genes are flanked upstream by a predicted integrase and downstream by a predicted transposase (Fig. 1A). Attachment (att) sites similar to those found in the *Vibrio* pathogenicity island 1 (VPI-1) also flank the region (41, 45).

To experimentally determine that the *tpeV* gene encodes a T6SS toxin, we engineered a Δ*tpeV* Δ*tpiV* target BGT49 strain (CC170). The Δ*tpeV* Δ*tpiV* target strain was then cocultured separately with the wild-type (WT) BGT49 killer, an isogenic Δ*tpeV* mutant (lacking the TpeV effector, CC167) and an isogenic Δ*vasK* nonkiller (T6SS^−^, CC168). The recovery of the Δ*tpeV* Δ*tpiV* target strain was significantly reduced (by approximately 5 orders of magnitude) when cocultured with wild-type BGT49 killer cells compared to the Δ*tpeV* or Δ*vasK* strains (Fig. 1B). This result suggests that TpeV is a T6SS effector that is actively used by V. cholerae strain BGT49 to eliminate susceptible cells that lack the TpiV immunity protein.

### The Aux 4 cluster can be expressed *in trans* in a clinical V. cholerae strain where it confers competitive advantages.

**FIG 2:**
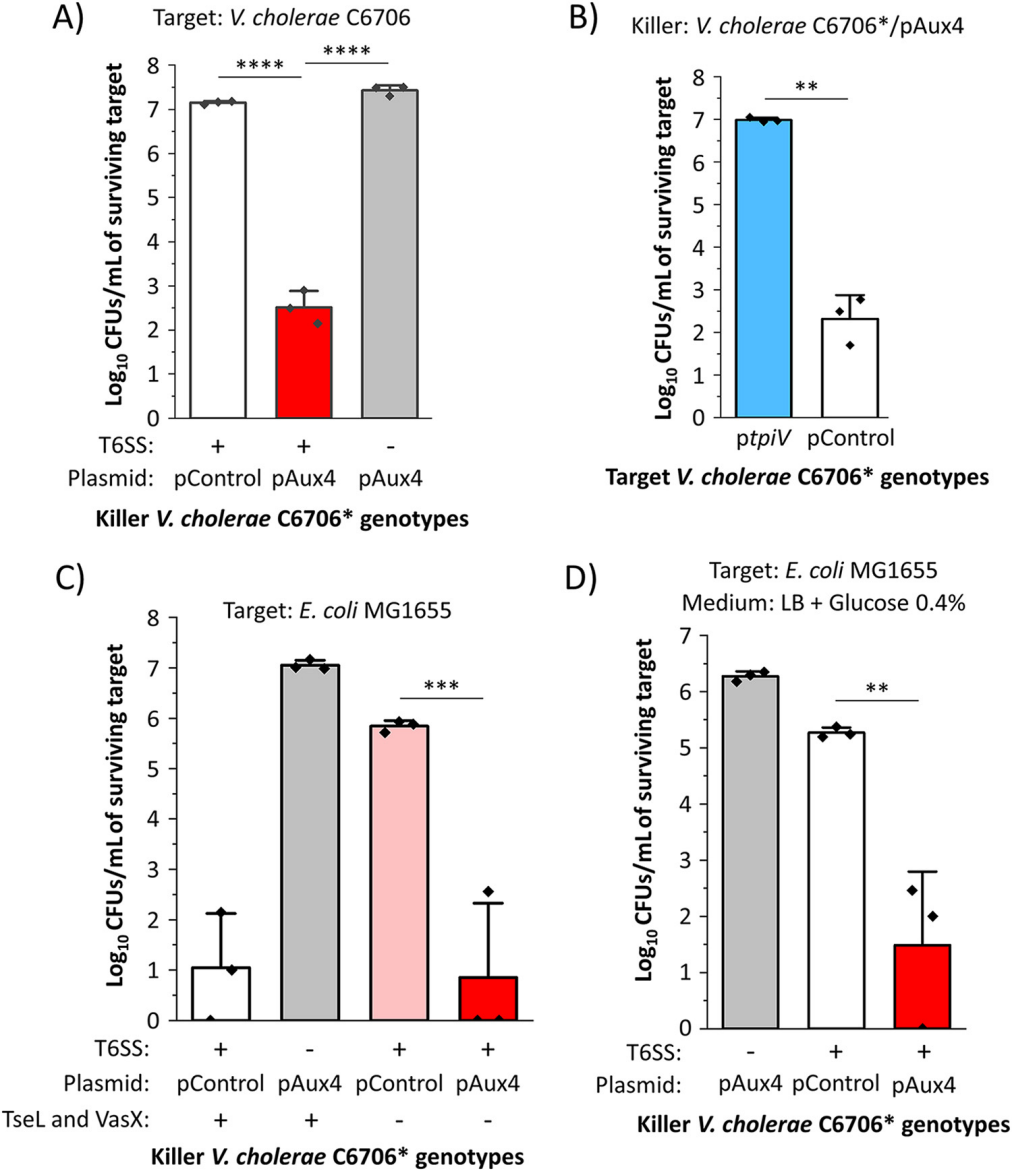
V. cholerae C6706* can use the Aux 4 cluster to eliminate target cells in a T6SS-dependent manner. (A) V. cholerae C6706* (T6SS^+^ or T6SS^−^) with a plasmid control or a plasmid encoding the Aux 4 cluster was cocultured with target parental V. cholerae C6706. A one-way ANOVA with a *post hoc* Tukey HSD test was used to determine significance. (B) Killer V. cholerae C6706* with the Aux 4 cluster was cocultured with target C6706 cells with a plasmid control or a plasmid encoding *tpiV*. Welch’s *t* test was used to determine significance. (C) V. cholerae C6706* with deletions in the known *tseL* and *vasX* T6SS effectors containing either a plasmid control or a plasmid with Aux 4 was cocultured with E. coli MG1655 cells. A one-way ANOVA with a *post hoc* Tukey HSD test was used to determine significance. (D) V. cholerae C6706* with a plasmid control or a plasmid encoding the Aux 4 cluster was cocultured with E. coli MG1655 on LB medium with 0.4% glucose. A one-way ANOVA with a *post hoc* Tukey HSD test was used to determine significance. ****, *P* < 0.0001; ***, *P* < 0.001; **, *P* < 0.01.

Since we observed that the Aux 4 cluster is located on a predicted mobile genetic element, we hypothesized that it can be used by other V. cholerae strains to eliminate competitor cells in a T6SS-dependent manner. In V. cholerae C6706, the QstR protein is a gene regulator that is required and sufficient to induce expression of T6SS genes (46–48). We cloned the Aux4 *vgrG*, *tap*, *tpeV*, *tpiV*, and *paar* genes on a plasmid (pAux4) under the control of the P*tac* promoter. We then introduced the pAux4 plasmid into V. cholerae strain C6706*, which constitutively expresses the QstR protein but does not possess Aux 4 cluster genes on its chromosomes (33, 46–48). The V. cholerae C6706* killer with the Aux 4 cluster on a plasmid (C6706*/pAux4) efficiently eliminates the parental target strain, unlike a killer C6706* strain carrying a plasmid control (Fig. 2A). By contrast, a C6706*/pAux4 T6SS^−^ strain cannot eliminate the parental target strain (Fig. 2A). To provide further evidence that TpiV can confer immunity, we introduced the *tpiV* gene into target V. cholerae C6706 and cocultured the strain with killer C6706*/pAux4 cells. V. cholerae C6706*/pAux4 kills V. cholerae target cells with a plasmid control but not when they encode the *tpiV* gene (Fig. 2B).

We next inquired whether the Aux 4 cluster can be used by V. cholerae to kill other target bacterial species. A C6706* strain that lacks the native TseL and VasX effectors poorly eliminates E. coli cells compared to a C6706* strain that harbors both toxins (Fig. 2C) (49, 50). However, the introduction of the Aux 4 cluster into the C6706* strain lacking TseL and VasX effectors restores its ability to efficiently eliminate E. coli cells (Fig. 2C). We recently reported that target E. coli cells are protected against T6SS attacks from strain C6706* when cocultured on LB medium supplemented with 0.4% glucose (4). By contrast, we observed that killer C6706*/pAux4 cells bypass the glucose-mediated resistance and efficiently eliminate E. coli cells when the coculture is performed on LB medium with glucose (Fig. 2D). These results confirm that the Aux 4 cluster can be used by V. cholerae to intoxicate competitor cells.

### TpeV permeabilizes cells and disrupts the membrane potential.

**FIG 3:**
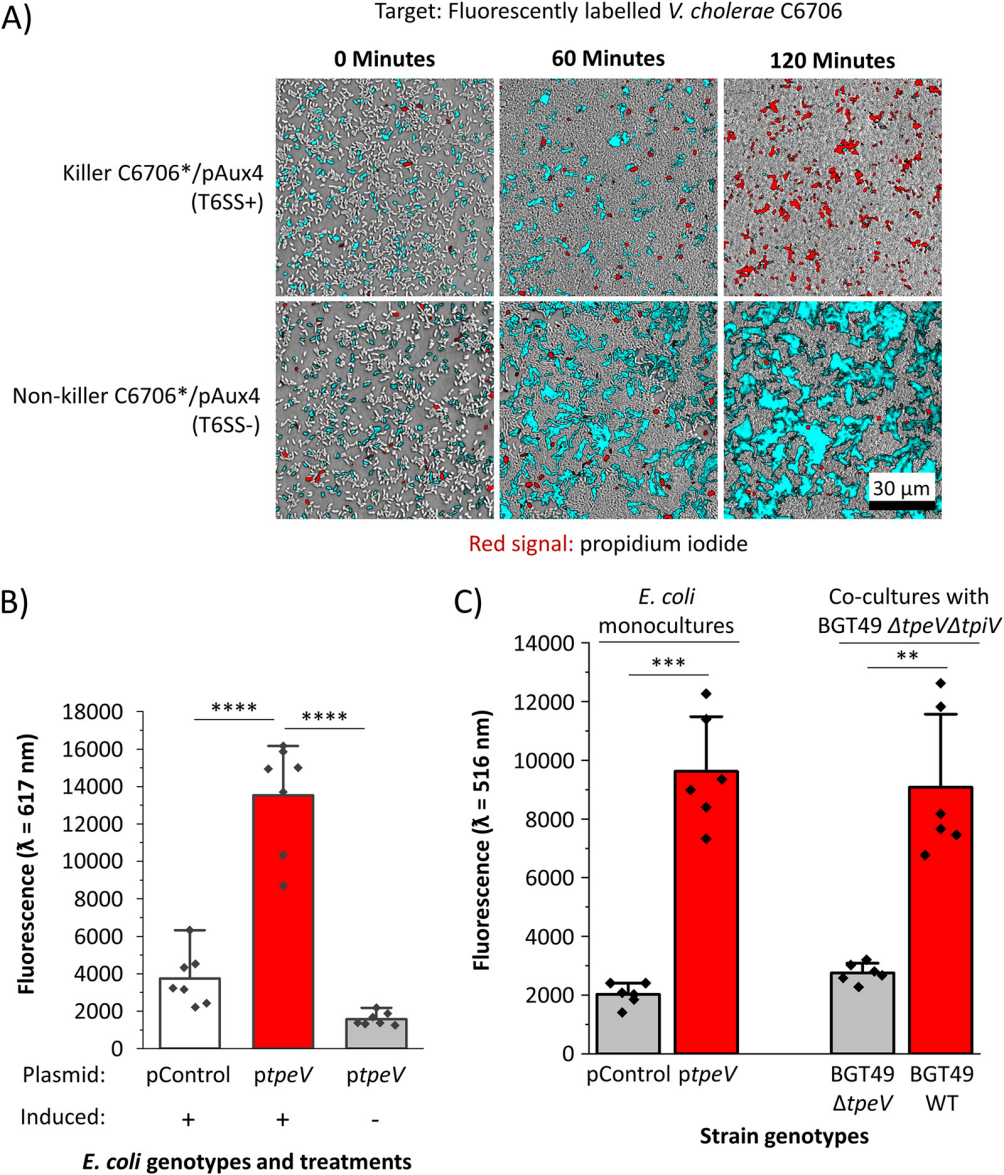
TpeV permeabilizes target cells and disrupts the membrane potential, leading to cytotoxicity. (A) Confocal microscopy was used to visualize a coculture between C6706* cells with Aux 4 (T6SS^−^ or T6SS^+^) and fluorescently labeled target C6706 cells in the presence of propidium iodide. Scale bar = 30 μm. (B) E. coli cells carrying a periplasmic *tpeV* construct or plasmid control were incubated with propidium iodide. Fluorescence readings were taken at an excitation ƛ of 535 nm and emission ƛ of 617 nm. A one-way ANOVA with a *post hoc* Tukey HSD test was used to determine significance. (C) E. coli cells carrying a periplasmic *tpeV* construct or plasmid control, or V. cholerae BGT49 cocultures between target Δ*tpeV* Δ*tpiV* and wild-type or Δ*tpeV* killer cells were incubated with the membrane potential-sensitive DiBAC_4_(3) dye. Fluorescence readings were taken at an excitation ƛ of 490 nm and emission ƛ of 516 nm. Welch’s *t* tests were used to determine significance. ****, *P* < 0.0001; ***, *P* < 0.001; **, *P* < 0.01.

We used confocal microscopy to examine cocultures between fluorescently labeled target V. cholerae C6706 cells (shown as cyan) and unlabeled killer C6706*/pAux4 cells (Fig. 3A). To each coculture, we added propidium iodine (PI), a molecule that cannot penetrate cells with intact membranes but exhibits high fluorescence when bound to the DNA of cells with compromised membranes. Fluorescently labeled V. cholerae target cells are successfully eliminated when cocultured with killer C6706*/pAux4 cells but remain viable when competing cells cannot assemble the T6SS apparatus (T6SS^−^) (Fig. 3A). Furthermore, a robust PI signal (depicted with red) is detectable when target cells are cocultured with C6706*/pAux4 cells (Fig. 3A). These results provide evidence that V. cholerae harboring the Aux 4 cluster can permeabilize target cells in a T6SS-dependent manner.

We next sought to further characterize the activity of the TpeV effector. The protein does not share primary sequence homology to known toxins and does not contain motifs or domains indicative of its toxicity. Tertiary structural prediction algorithms also fail to detect significant homologs with known functions. TpeV has 11 cysteine residues, suggesting that multiple disulfide bonds could play roles in stabilizing the protein. Transmembrane prediction algorithms TMHMM and Phobius do not detect extensive transmembrane regions, and SignalP 5.0 does not predict a signal sequence (see Fig. S1 and S2 in the supplemental material) (51–53). We also attempted to identify TpeV homologs using the secondary structure predictor JPred (54). While most homologs are hypothetical proteins with unknown functions, some contain domains similar to the peptidoglycan-binding C-terminal regions of the OmpA protein (55, 56). OmpA proteins are involved in pathogenesis and have diverse functions that include formation of porins and channels (57, 58). Because target V. cholerae cells have a substantial PI signal when cocultured with killer cells harboring the Aux 4 cluster, we hypothesized that TpeV might permeabilize target cells when delivered to the periplasm.

10.1128/mSphere.00318-21.1 FIG S1 TpeV Phobius transmembrane helix prediction. The amino acid sequence of TpeV was analyzed using the Phobius transmembrane predictor. Download FIG S1, TIF file, 0.9 MB. Copyright © 2021 Crisan et al. 2021 Crisan et al. https://creativecommons.org/licenses/by/4.0/ This content is distributed under the terms of the Creative Commons Attribution 4.0 International license.

10.1128/mSphere.00318-21.2 FIG S2 TpeV SignalP prediction. The amino acid sequence of TpeV was analyzed using the SignalP 5.0 transmembrane predictor. Download FIG S2, TIF file, 1.6 MB. Copyright © 2021 Crisan et al. 2021 Crisan et al. https://creativecommons.org/licenses/by/4.0/ This content is distributed under the terms of the Creative Commons Attribution 4.0 International license.

To test this prediction, we introduced plasmid-borne *tpeV* with a periplasmically directing *pelB* sequence under the control of an inducible promoter into E. coli cells. A significantly higher PI signal is detected when E. coli cells are induced to express periplasmic TpeV compared to that of cells that harbor a plasmid control (Fig. 3B). We also hypothesized that TpeV disrupts the bacterial cell membrane potential (28, 59, 60). To test this hypothesis, we used the bis-(1,3-dibutylbarbituric acid) trimethine oxonol [DiBAC_4_(3)] potential-sensitive dye, which is excluded from cells with a normal membrane potential but exhibits fluorescence in depolarized cells (60–62). E. coli cells that express periplasmically delivered TpeV have significantly higher DiBAC_4_(3) uptake than E. coli cells that express a plasmid control (Fig. 3C). As a positive membrane depolarization control, we exposed E. coli harboring plasmid control or TpeV to the carbonyl cyanide *m*-chlorophenyl hydrazone (CCCP) ionophore (see Fig. S3 in the supplemental material). E. coli cells expressing periplasmically delivered TpeV have comparable DiBAC_4_(3) fluorescence to E. coli cells treated with CCCP (Fig. 3C; see also Fig. S3). To probe whether TpeV can disrupt the membrane potential in a T6SS-dependent manner, we cocultured V. cholerae BGT49 Δ*tpeV* Δ*tpiV* (CC170) target cells with either the BGT49 wild-type or BGT49 Δ*tpeV* (CC167) strain. Following cocultures with the wild-type but not the Δ*tpeV* strain, bacterial membrane potentials are disrupted as cells display an elevated DiBAC_4_(3) signal (Fig. 3C). Taken together, these findings demonstrate that TpeV permeabilizes cells and disrupts the membrane potential of target bacteria.

10.1128/mSphere.00318-21.3 FIG S3 CCCP disrupts the membrane potential of E. coli cells. E. coli cells harboring either plasmid control or periplasmically delivered TpeV were first incubated with CCCP and then with the membrane potential-sensitive DiBAC_4_(3) dye. Fluorescence readings were taken at an excitation ƛ = 490 nm and emission ƛ = 516 nm. A one-way ANOVA was used to determine significance. NS, not significant. Download FIG S3, TIF file, 0.9 MB. Copyright © 2021 Crisan et al. 2021 Crisan et al. https://creativecommons.org/licenses/by/4.0/ This content is distributed under the terms of the Creative Commons Attribution 4.0 International license.

### TpeV belongs to a large family of T6SS proteins and is spread widely across V. cholerae isolates.

**FIG 4:**
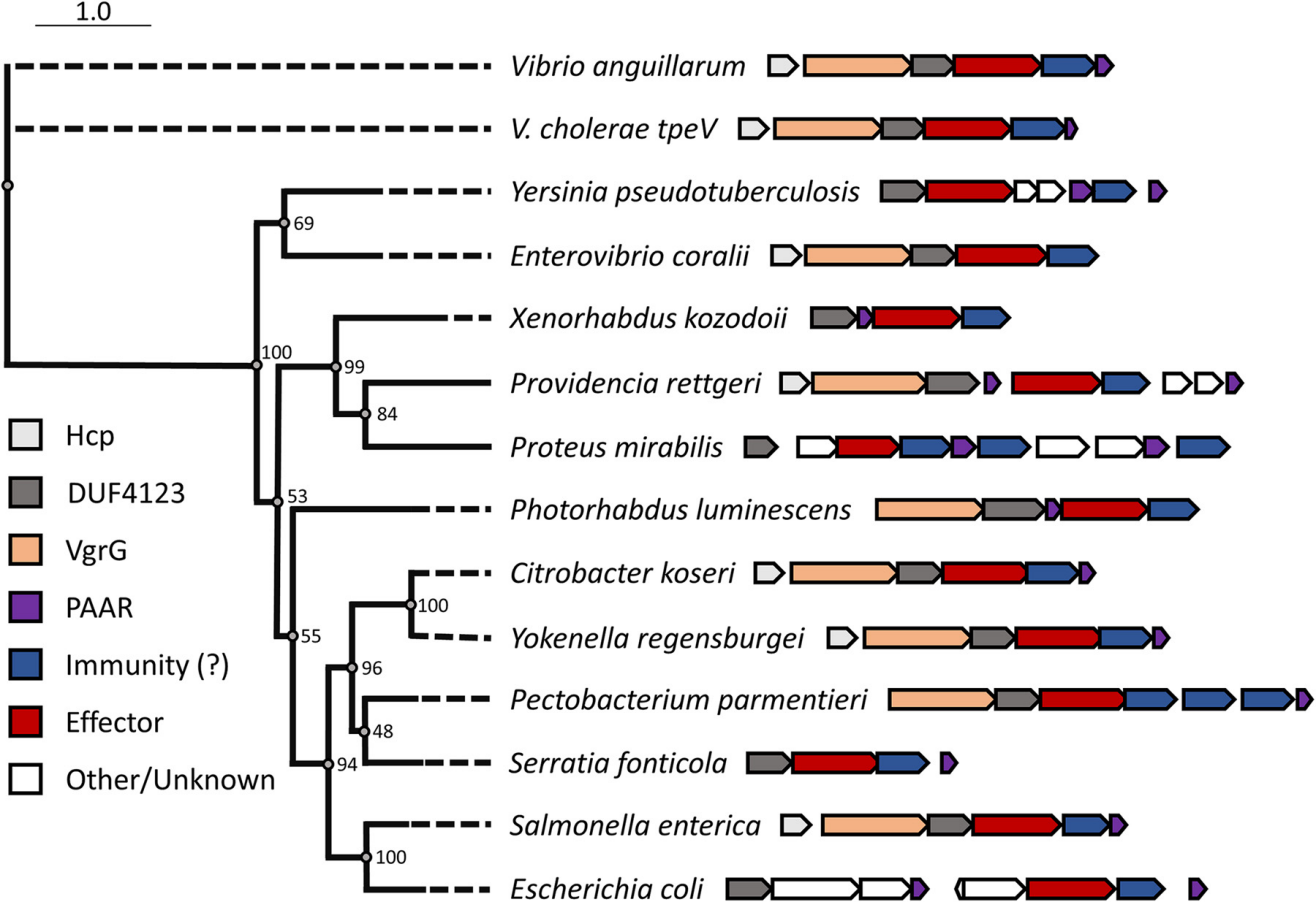
*TpeV* homologs are found in many bacterial species near other T6SS genes. TpeV homologs were identified using PHMMER, and selected sequences were aligned using MUSCLE. A phylogenetic tree was constructed with 100 bootstraps.

Since the sequence or predicted structure of TpeV shares no homology to known toxins (including known permeabilizing toxins), we used PHMMER to search for homologs in other bacterial species (63). We identified *tpeV*-like genes across diverse *Gammaproteobacteria* (Fig. 4; see also Table S1 in the supplemental material). In all selected species, genes coding for TpeV homologs are found near known T6SS genes like *hcp*, *vgrG*, DUF4123-containing chaperones, or other structural components (Fig. 4). Furthermore, we observed that *tpeV-tpiV* sequences are present in both patient and environmental V. cholerae genomes and are broadly distributed in countries across Africa, Asia, Europe, North America, and South America (see Table S2 in the supplemental material). Our results indicate that TpeV is a representative member of a family of T6SS toxins with antimicrobial activity that allows cells to eliminate competitor bacteria.

10.1128/mSphere.00318-21.6 TABLE S1 Complete list of all identified TpeV protein homologs using the PHMMER algorithm. Download Table S1, XLS file, 0.05 MB. Copyright © 2021 Crisan et al. 2021 Crisan et al. https://creativecommons.org/licenses/by/4.0/ This content is distributed under the terms of the Creative Commons Attribution 4.0 International license.

10.1128/mSphere.00318-21.7 TABLE S2 V. cholerae strains from diverse geographical locations harbor *tpeV-tpiV* modules. Download Table S2, DOCX file, 0.02 MB. Copyright © 2021 Crisan et al. 2021 Crisan et al. https://creativecommons.org/licenses/by/4.0/ This content is distributed under the terms of the Creative Commons Attribution 4.0 International license.

## DISCUSSION

Here, we show that many bacterial species encode homologs of a previously undescribed T6SS protein that intoxicates, permeabilizes, and disrupts the membrane potential of target cells. While studies have examined antibacterial effectors from clinical V. cholerae isolates, such as C6706 and V52, we and others found that strains isolated from sources other than patients encode a more diverse set of putative T6SS toxins (8, 28, 31, 34, 35, 41). Many T6SS toxins degrade components of the cell envelope; lipases and peptidoglycan-degrading enzymes target lipid membranes and cell walls, respectively (6, 30, 32, 49, 64).

Pore-forming proteins represent another functional class of T6SS toxins that act in the bacterial cell envelope (28, 59, 60, 65). The V. cholerae VasX effector encoded in the Aux 2 cluster is a large protein that contains a C-terminal colicin domain effective at eliminating both bacterial and eukaryotic cells (28, 29). Since it is predicted to form large pores, VasX permeabilizes cells and allows passage of molecules like PI into the cell (28). The Pseudomonas aeruginosa Tse4 and the Serratia marcescens Ssp6 effectors are both relatively small proteins that form ion-selective pores but do not allow larger molecules like PI to enter cells (59, 60). Recently, Vibrio parahaemolyticus has also been shown to harbor T6SS effectors that disrupt cellular membranes (65).

Importantly, the V. cholerae TpeV T6SS effector that we describe in this study does not contain known features, and its sequence does not share homology to any previously characterized proteins. We provide evidence that TpeV is a T6SS toxin that can be used by V. cholerae cells to permeabilize target cells and disrupt the cell membrane potential (Fig. 2 and 3). The cell membrane potential is essential for ATP synthesis, cell division, and membrane transport (66–68). Therefore, TpeV-mediated toxicity is likely to inflict substantial damage to target cells by perturbing multiple essential processes.

We hypothesize that TpeV could permeabilize cells by forming pores. Pore-forming toxins (PFTs) are widespread among all kingdoms of life (69–73). Based on the secondary structure of the membrane-spanning domain, two major classes of PFTs have been described: α-PFTs and β-PFTs (69, 71, 74). α-PTFs include the E. coli colicin and cytolysin A families, while β-PFTs are found in many Gram-positive bacterial species and contribute to the virulence of pathogens like Staphylococcus aureus and Clostridium perfringens (71, 73–75). Our homology predictions suggest that TpeV might harbor a peptidoglycan-binding OmpA-like domain (55). RmpM is a Neisseria meningitidis periplasmic protein that also possesses an OmpA-like domain (56). Experimental evidence suggests that RmpM stabilizes oligomeric porins in the outer membrane (56, 76). Rather than form new pores, it is also possible that TpeV might interact with and disrupt the normal functions of existing porins or channels in the membranes of target bacteria. Future experiments will determine whether TpeV forms pores or employs other mechanisms that damage membranes and permeabilize cells.

In strain BGT49, the Aux 4 cluster and a restriction modification system are found near a prophage integrase and a transposase (Fig. 1A). This suggests that the genes are located on a mobile genetic element that can be transferred between bacterial cells to confer advantages against phages and other bacteria (41). The V. cholerae T6SS Aux 3 cluster was also experimentally validated to be located on a mobile genetic element (37). Our results show that V. cholerae strain C6706* can use the Aux 4 cluster to kill parental cells and support the hypothesis that the Aux 4 cluster can be transferred to confer competitive advantages. This hypothesis is further supported by our observation that TpeV homologs are found close to other T6SS genes in many bacterial species, including human pathogens (Providencia rettgeri, Proteus mirabilis, Citrobacter koseri, Yokenella regensburgei, Serratia fonticola, Salmonella enterica, and E. coli), animal pathogens (Vibrio anguillarum and Photorhabdus luminescens), and plant pathogens (Pectobacterium parmentieri) (77–84) (Fig. 4). TpeV homologs found in other bacteria are also located near transposase-like genes (data not shown).

We also hypothesized that the *paar* gene downstream of *tpiV* might be required for *tpeV*-mediated toxicity (Fig. 1). However, we observed that a BGT49 strain with an Aux 4 *paar* deletion (Δ*paar*, CC179) eliminated susceptible Δ*tpeV* Δ*tpiV* cells in a similar manner to wild-type BGT49, suggesting that the PAAR protein is not required for TpeV-mediated killing (see Fig. S4 in the supplemental material). All known T6SS toxic effectors are neutralized by cognate immunity proteins, which are generally encoded by genes adjacent to effectors (28, 85). We found that *tpiV*, the gene immediately downstream of *tpeV*, confers immunity to target cells against *tpeV-*mediated toxicity (Fig. 1B and 2B). SignalP predicts that TpiV encodes a periplasmic Sec-tag, which is expected since TpeV exhibits its toxicity when delivered to the periplasm of target cells (Fig. 3B and C; see also Fig. S5 in the supplemental material). In other species, we observed that multiple putative immunity proteins can be found near TpeV homologs (Fig. 4). Additional studies are required to confirm which predicted TpiV-like proteins are the cognate immunity factors for the TpeV homologs.

10.1128/mSphere.00318-21.4 FIG S4 The Aux 4 *paar* gene is not required for TpeV-mediated killing. Target V. cholerae BGT49 Δ*tpeV*Δ*tpiV* (CC170) was cocultured with either Δ*paar* (CC179), WT, Δ*vasK* (T6SS^−^, CC168), or Δ*tpeV* (CC167) BGT49. Download FIG S4, TIF file, 1.8 MB. Copyright © 2021 Crisan et al. 2021 Crisan et al. https://creativecommons.org/licenses/by/4.0/ This content is distributed under the terms of the Creative Commons Attribution 4.0 International license.

10.1128/mSphere.00318-21.5 FIG S5 TpiV SignalP prediction. The amino acid sequence of TpiV was analyzed using the SignalP 5.0 transmembrane predictor. Download FIG S5, TIF file, 1.6 MB. Copyright © 2021 Crisan et al. 2021 Crisan et al. https://creativecommons.org/licenses/by/4.0/ This content is distributed under the terms of the Creative Commons Attribution 4.0 International license.

In conclusion, we demonstrate that the T6SS Aux 4 cluster found in many V. cholerae isolates encodes a toxin that can be used to eliminate competitor bacteria. TpeV is a T6SS effector that permeabilizes target bacteria and disrupts the membrane potential, leading to severe cellular intoxication. However, target cells expressing TpiV are protected and resist TpeV-mediated toxicity. Finally, we find that TpeV homologs are widespread among Gram-negative bacteria, suggesting that the protein represents a novel and potent antimicrobial agent of interest for further studies. Understanding the molecular mechanisms of antimicrobial toxins that drive competition and antagonism could lead to the development of novel biotechnology and medical applications.

## MATERIALS AND METHODS

### Bacterial strains and plasmids.

Plasmids were constructed using standard molecular biology techniques. Gibson mix reagents, restriction enzymes, and polymerases were used as recommended by manufacturers (Promega and New England BioLabs). Plasmids were verified by PCR and Sanger sequencing (Eurofins and Eton Bioscience). V. cholerae C6706 mutant strains were made using pKAS allelic exchange methods as described previously (86). V. cholerae BGT49 mutant strains were made using natural transformation as described previously with modifications (87, 88). Briefly, overnight cultures were back-diluted in fresh LB medium for approximately 1 h and then statically incubated overnight at 30°C in liquid LB medium with a sterile crab shell fragment. Crab shells were transferred to fresh LB medium containing 30 to 50 μg of a plasmid engineered to encode ∼1,000-bp flanking regions to replace the desired genes with an antibiotic cassette. Cells were incubated statically overnight at 30°C and then spread on antibiotic plates to select for transformants. BGT49 mutants were confirmed by PCR and antibiotic resistance. Bacterial strains and plasmids used are listed in Table S3 in the supplemental material.

10.1128/mSphere.00318-21.8 TABLE S3 Strains and plasmids used in this study. Download Table S3, DOCX file, 0.02 MB. Copyright © 2021 Crisan et al. 2021 Crisan et al. https://creativecommons.org/licenses/by/4.0/ This content is distributed under the terms of the Creative Commons Attribution 4.0 International license.

### Bacterial competition assays.

Bacterial cultures were grown overnight in liquid LB medium at 37°C with shaking. Overnight cultures were back-diluted and incubated in liquid LB medium at 37°C with shaking for 3 h. Bacterial cultures were then normalized to an optical density at 600 nm (OD_600_) of 1. If strains harbored plasmids, cultures were grown overnight with antibiotics to maintain plasmids and 100 μM isopropyl-β-d-thiogalactopyranoside (IPTG) if plasmids contained an inducible promoter. If strains were grown in medium containing antibiotics, liquid cultures were then washed three times with fresh LB medium before they were cocultured. For bacterial competitions performed on LB agar medium with 0.4% glucose, overnight and back-diluted cultures were also grown in LB medium supplemented with 0.4% glucose. A 50-μl mixture aliquot of a 10:1 killer/target cell ratio was spotted on a 0.22-μm-pore-size filter paper, which was placed on LB agar medium and incubated at 37°C. After 3 h, filters were vortexed in 5 ml of sterile LB medium for 30 s. One hundred microliters of serial dilutions were then spread (or 3 μl or serial dilutions were spotted) on plates containing the required antibiotic to select for target cells. Data from three cocultures were used to determine significance. Results are representative of at least two independent experiments.

### Confocal microscopy.

Overnight cultures were back-diluted 1:100 for 3 h in liquid LB medium. Samples were then normalized to an OD_600_ of 1. A 2-μl aliquot of 10:1 killer/target cell mixture was spotted on top of a dry 8-μl aliquot of propidium iodide (100 μg/ml) on an LB agar pad. A Nikon A1R confocal microscope using a Perfect Focus System with a 40× objective (Plan Fluor ELWD 40× DIC M N1) was used to stabilize the focus in the plane of the colony growth. Cells were imaged at 90 to 100% humidity and 37°C. Images were processed using ImageJ. Results are representative of at least three independent experiments.

### Membrane permeabilization assays.

Bacterial cultures of E. coli Shuffle T7 Express (New England BioLabs) cells carrying either a control plasmid or a periplasmic *tpeV* construct were grown overnight in liquid LB medium supplemented with 0.2% glucose and ampicillin at 37°C with shaking. Cells were washed three times with LB and 100× back-dilutions were made in fresh liquid LB medium with 500 μM IPTG (or 0.2% glucose for uninduced controls) and ampicillin. Strains were incubated at 37°C for 2 h, washed three times with phosphate-buffered saline (PBS), and normalized to an OD_600_ of 1. One hundred microliters of each culture was incubated with 1 μl propidium iodide (1 mg/ml) for 15 to 30 min. Fluorescence values were taken on a Synergy BioTek plate reader using an excitation ƛ of 535 nm and emission ƛ of 617 nm and normalized by subtracting the average values from samples with propidium iodide but no cells. Data represent the averages obtained from seven biological replicates from two independent experiments.

### Membrane potential assays.

Bacterial cultures of E. coli Shuffle T7 Express (New England BioLabs) cells carrying either a control plasmid or a periplasmic *tpeV* construct were grown overnight with shaking at 37°C in liquid LB medium supplemented with 0.2% glucose and ampicillin. Cells were washed three times with LB, and 100× back-dilutions were incubated at 37°C for 2 h in fresh liquid LB medium with 500 μM IPTG and ampicillin. Cells were again washed three times with PBS and normalized to an OD_600_ of 1 in PBS. Cells were incubated for 30 min in the dark with DiBAC_4_(3) at a final concentration of 10 μM and washed three times with PBS. For positive depolarization controls, E. coli cells were incubated with 10 μM carbonyl cyanide *m*-chlorophenyl hydrazone for 10 min in the dark prior to staining with DiBAC_4_(3). Fluorescence values were taken on a Synergy BioTek plate reader using an excitation ƛ of 490 nm and emission ƛ of 516 nm. Data represent the averages obtained from six biological replicates from two independent experiments.

For coculture measurements of membrane potentials, overnight cultures of the indicated V. cholerae BGT49 strains were back-diluted and incubated in liquid LB medium at 37°C with shaking for 3 h. Bacterial cultures were then normalized to an OD_600_ of 10. A 50-μl mixture aliquot with a ratio of 1:1 killer/target cells was spotted on a 0.22-μm-pore-size filter paper, which was placed on LB agar medium and incubated at 37°C. After 2 h, filters were vortexed in sterile LB medium for 30 s. Cells were washed three times with PBS, normalized to an OD_600_ of 1, incubated for 30 min in the dark with DiBAC_4_(3) at a final concentration of 10 μM and washed three times with PBS. Fluorescence values were taken on a Synergy BioTek plate reader using an excitation ƛ of 490 nm and emission ƛ of 516 nm. Data represent the averages obtained from six biological replicates from two independent experiments.

### Bioinformatic analyses.

The PHMMER server was used to search for homologs of TpeV in the UniProtKB database (63, 89). Obsolete or duplicate hits were removed from Table S1. Selected homologs were aligned using MUSCLE and were used to retrieve genomes from the NCBI database (90). A phylogenetic tree was constructed using PhyML with 100 bootstrap values and visualized using PRESTO (91–93). Putative immunity proteins were predicted based on homology to TpiV and genomic location. Truncated *vgrG*-like genes encoding stop codons were observed in some species but were excluded from Fig. 4.

The *tpeV-tpiV* genes were used to retrieve homologous sequences from the NCBI RefSeq Genome Database (94). Selected strains harboring homologs to the *tpeV-tpiV* gene module are displayed in Table S2 in the supplemental material.
