# Supplementary figures and images for: A New Contact Killing Toxin Permeabilizes Cells and Belongs to a Broadly Distributed Protein Family
Source: mSphere. 2021 Jul 21;6(4):e00318-21. doi: 10.1128/mSphere.00318-21 (PMC8386463; doi:10.1128/mSphere.00318-21)

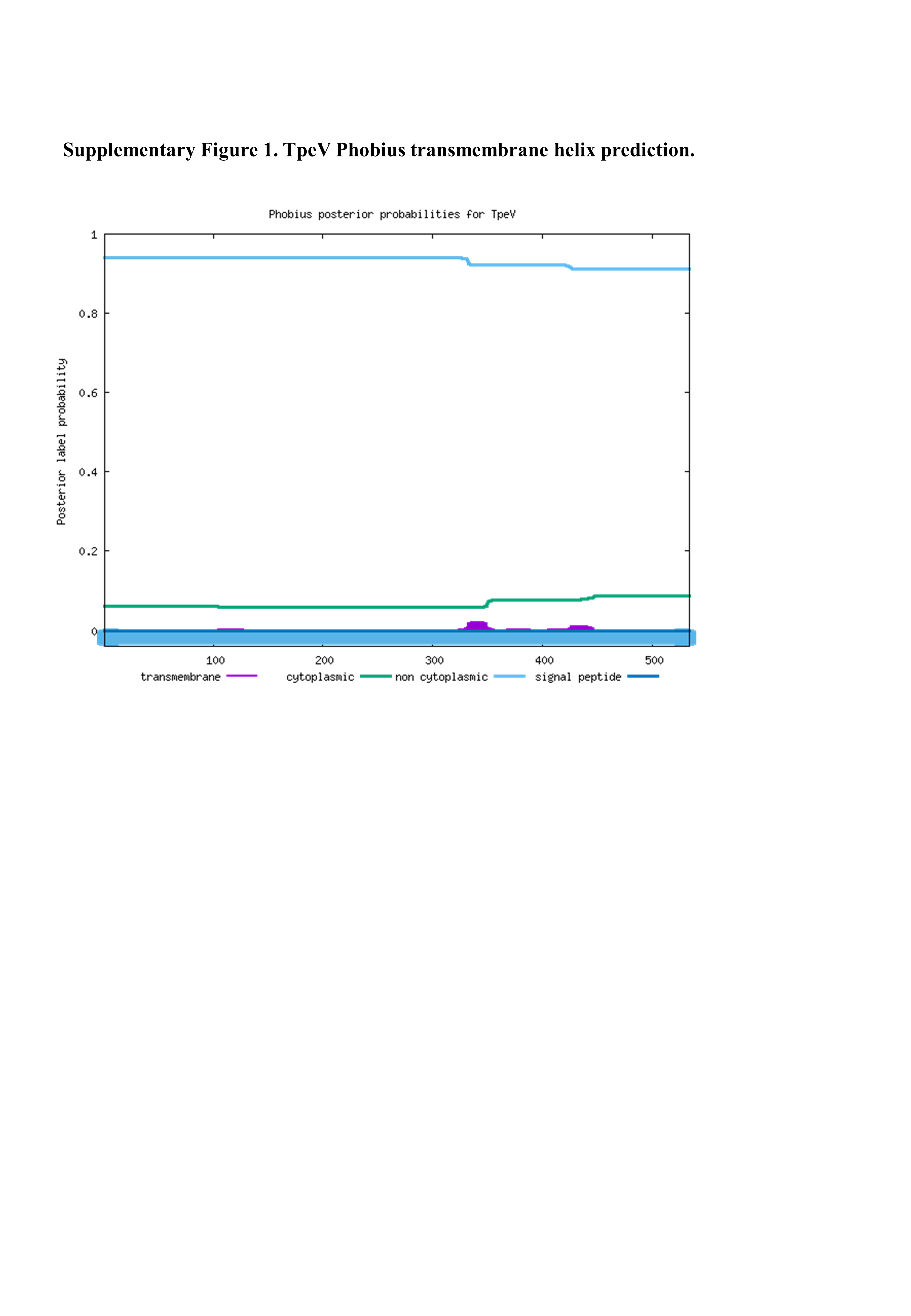

Supplement: FIG S1 [file msphere.00318-21-sf001.tif]

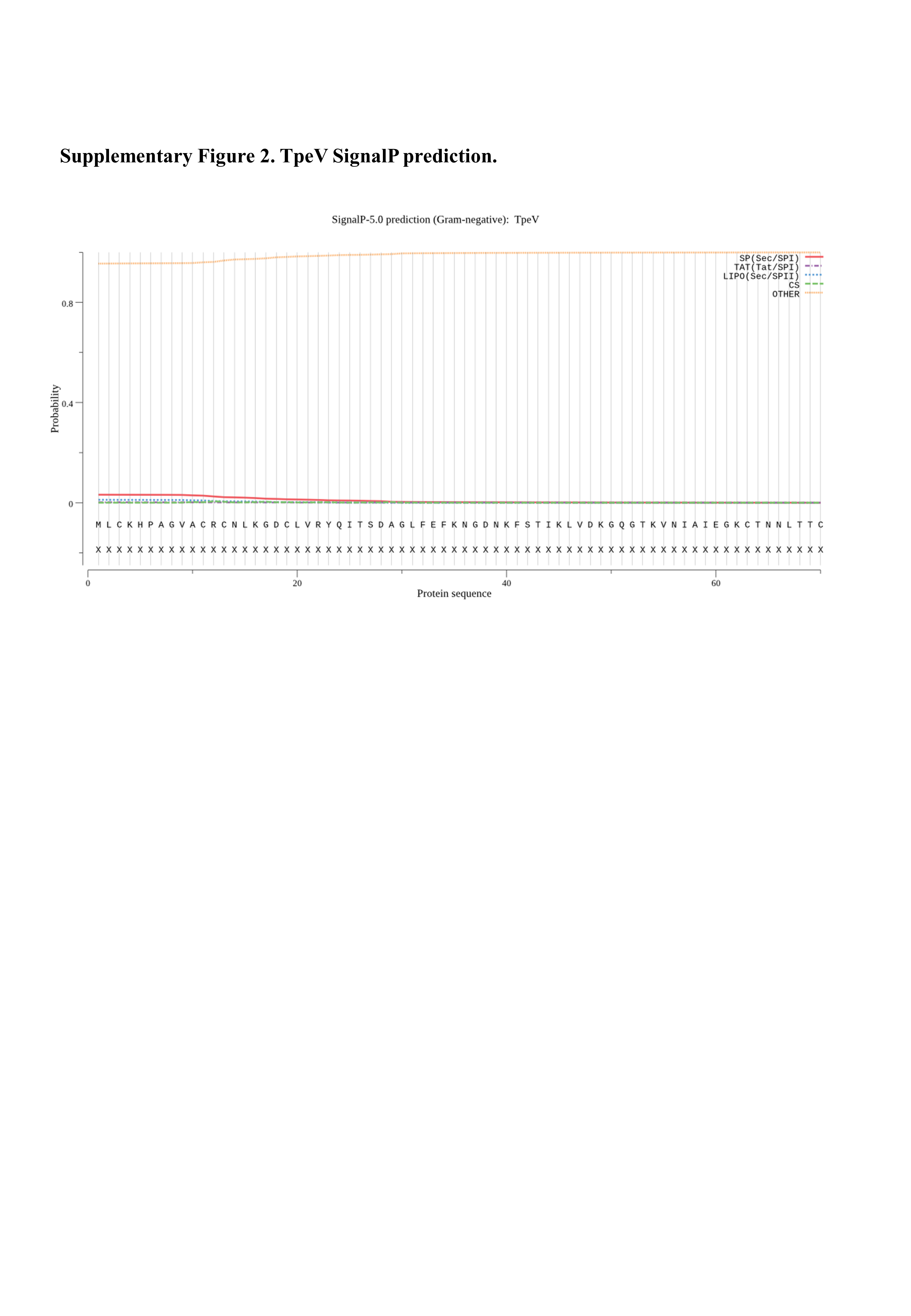

Supplement: FIG S2 [file msphere.00318-21-sf002.tif]

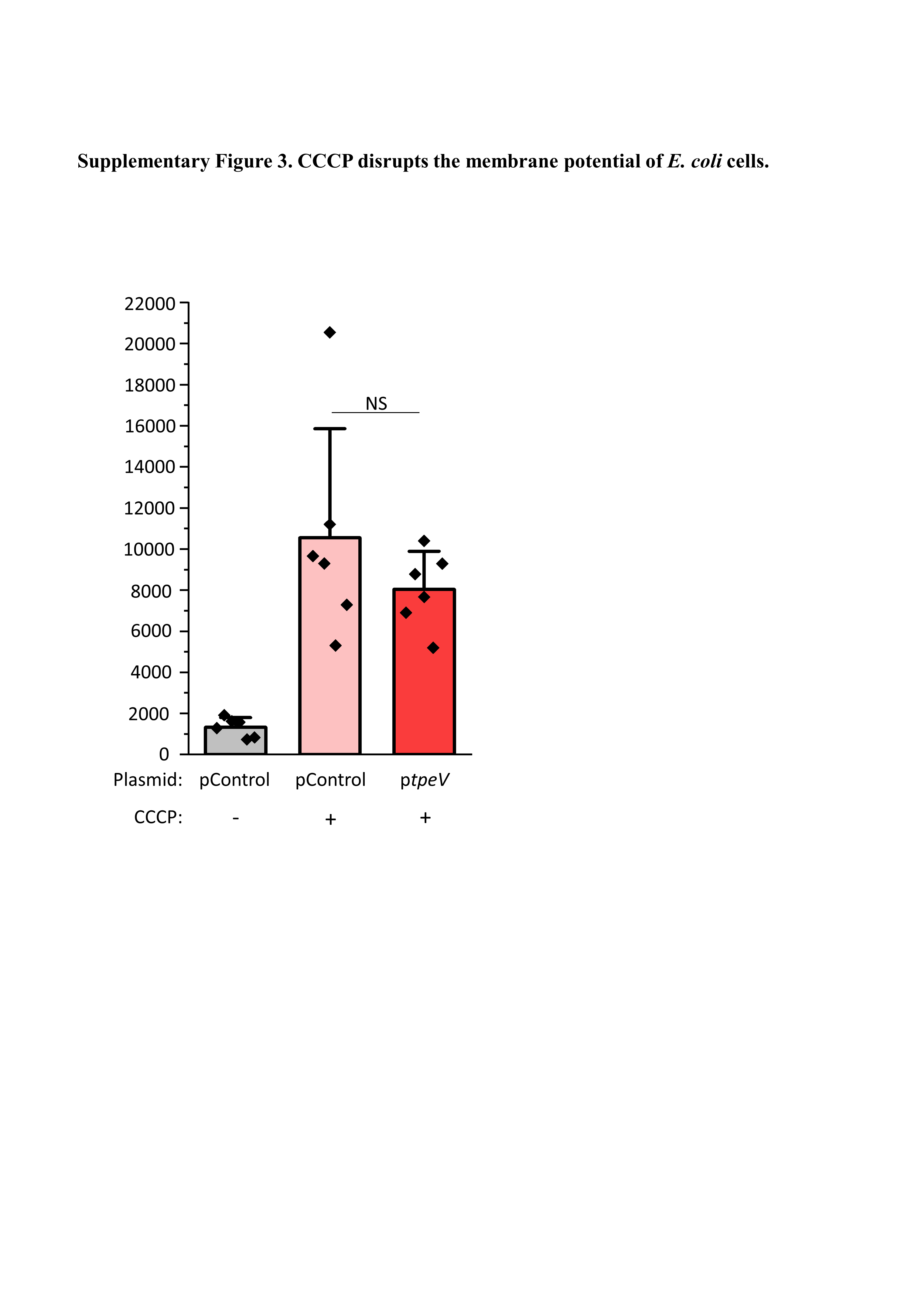

Supplement: FIG S3 [file msphere.00318-21-sf003.tif]

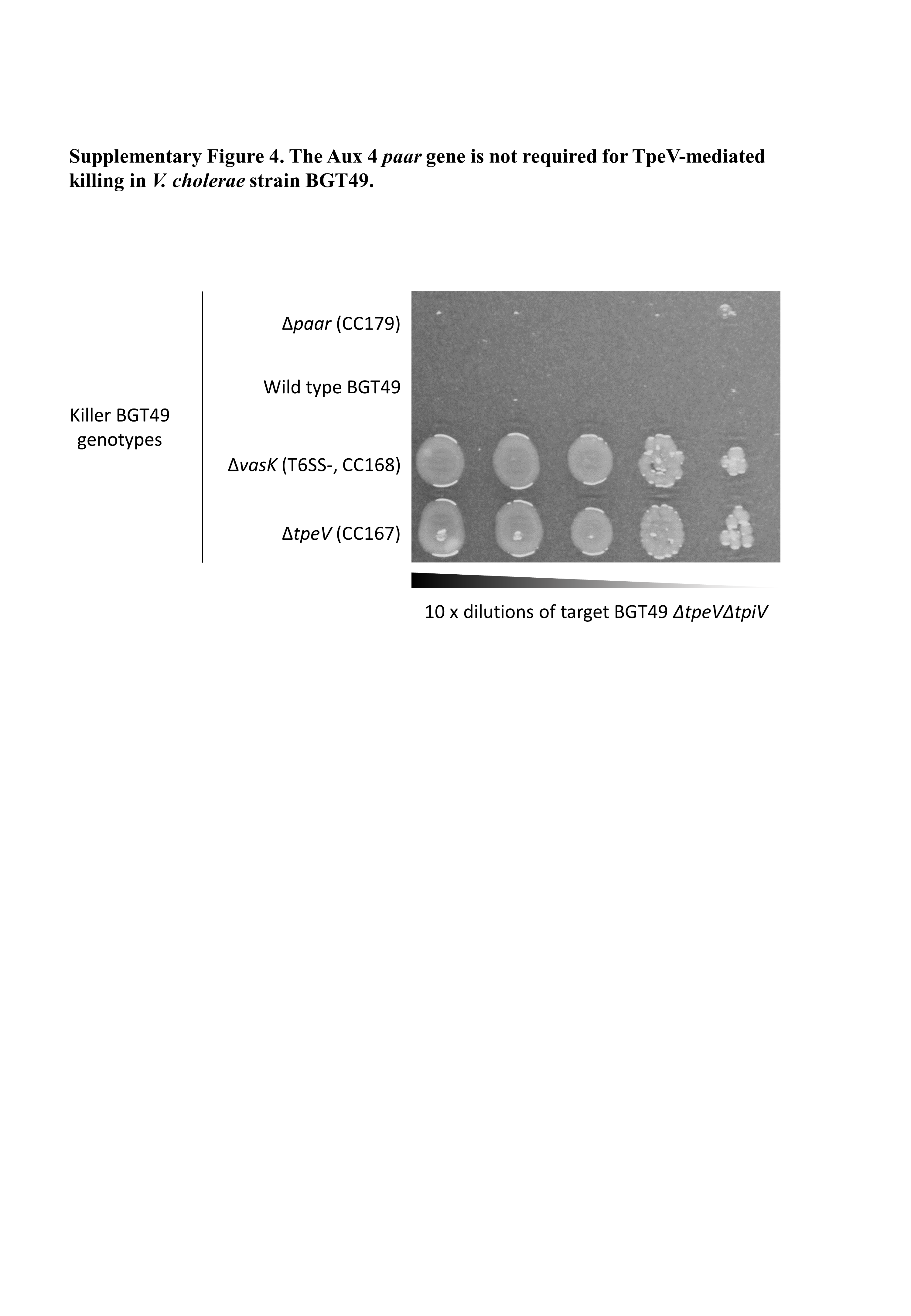

Supplement: FIG S4 [file msphere.00318-21-sf004.tif]

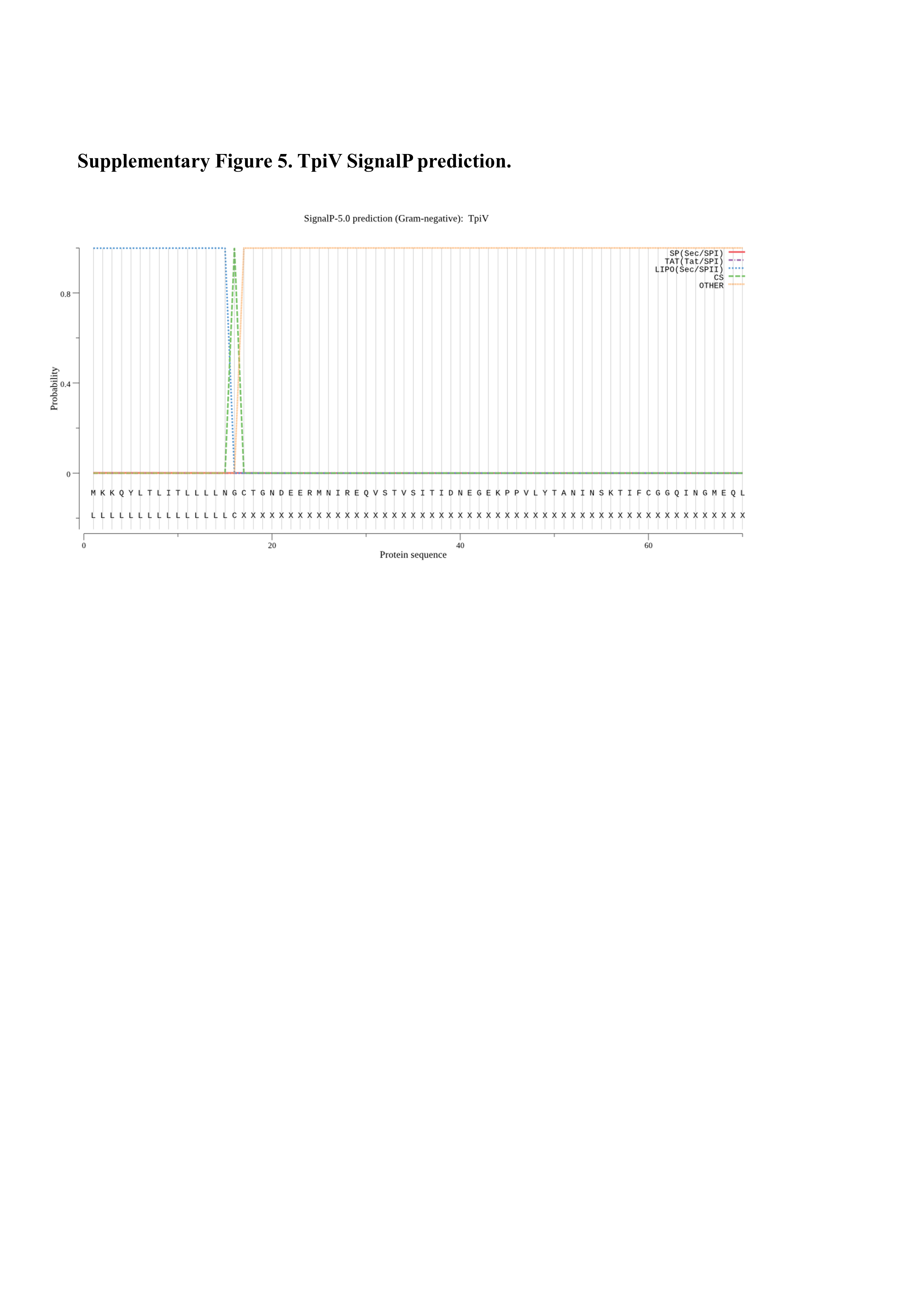

Supplement: FIG S5 [file msphere.00318-21-sf005.tif]
