# Supplementary material for: A New Contact Killing Toxin Permeabilizes Cells and Belongs to a Broadly Distributed Protein Family
Source: mSphere. 2021 Jul 21;6(4):e00318-21. doi: 10.1128/mSphere.00318-21 (PMC8386463; doi:10.1128/mSphere.00318-21)
Supplement: TABLE S2 [file msphere.00318-21-st002.docx]

| Strain | Geographic Location | Isolation Source |
| --- | --- | --- |
| 1421-77 | India | Diarrhea |
| 173V1015 | Bangladesh | Water |
| 2012Env-2 | Haiti | Water, Environmental |
| 234_03 | Brazil, Sao Paulo | Sewer |
| 5473-62 | Philippines | Diarrhea |
| 617 | Ukraine | Patient Feces |
| A110912Z1 | Austria, Neusiedler See | Zooplankton |
| A12JL4W93 | Austria, Neusiedler See | Water |
| BRV8 | United Kingdom | Blood culture |
| CHNf1 | China, Shanghai | Sea water |
| CISM_300055 | Mozambique, Manhica | Stool |
| DL4211 | USA, Mouth of Rio Grande | Water |
| FORC_076 | South Korea | Watery diarrhea |
| IDH-06787 | India, Kolkata | Stool |
| OYP6F09 | USA, Massachusetts | Oyster pond |
| P2-CHT15-00 | Austria, Neusiedler See | Patient with otitis externa |
| RFB05 | USA, Pittsburgh | North Park Lake |
| Vc306 | China, Shenzhen | Pork |

**Supplementary Table 2. *V. cholerae* strains from diverse geographical locations harbor *tpeV-tpiV* modules.**
