# Supplementary material for: A New Contact Killing Toxin Permeabilizes Cells and Belongs to a Broadly Distributed Protein Family
Source: mSphere. 2021 Jul 21;6(4):e00318-21. doi: 10.1128/mSphere.00318-21 (PMC8386463; doi:10.1128/mSphere.00318-21)
Supplement: TABLE S3 [file msphere.00318-21-st003.docx]

**Supplementary Table 3. Strains and plasmids used in this study.**

**Bacterial Strains**

| Strain Number | Strain Background | Genotype | Plasmid | Reference |
| --- | --- | --- | --- | --- |
| BGT49 | *V. cholerae* VC56 | WT |  | Bernardy et al. Appl. Env. Micro. 2016. |
| CC167 | *V. cholerae* VC56 | ∆*tpeV*::*kanR* |  | This study |
| CC168 | *V. cholerae* VC56 | ∆*vasK*::*kanR* |  | This study |
| CC170 | *V. cholerae* VC56 | ∆*tpeV*-*tpiV*::SpecR |  | This study |
| CC179 | *V. cholerae* VC56 | ∆*paar(Aux4)*::*kanR* |  | This study |
| JT516 | *V. cholerae* C6706 | *∆lacZ::SpecR* |  | Crisan et al. Genome Bio. 2019 |
| CC61 | *V. cholerae* C6706 | *ptac-qstR* | pEVS143*-Aux4* | This study |
| CC72 | *V. cholerae* C6706 | *ptac-qstR* | pEVS143 | This study |
| CC157 | *V. cholerae* C6706 | *ptac-qstR, ΔvasK* | pEVS143*-Aux4* | This study |
| KE06 | *V. cholerae* C6706 | *∆lacZ::SpecR* | pEVS143*-tpiV* | This study |
| CC153 | *V. cholerae* C6706 | *∆lacZ::SpecR* | pEVS143 | This study |
| HC17 | *V. cholerae* C6706 | *∆VC1807::ptac-mTFP1 Ptac-qstR ∆tseL ∆tsiV1 ∆vasX ∆tsiV2* | pEVS143*-Aux4* | This study |
| CC160 | *V. cholerae* C6706 | *∆VC1807::ptac-mTFP1 Ptac-qstR ∆tseL ∆tsiV1 ∆vasX ∆tsiV2* | pEVS143 | This study |
| SN440 | *V. cholerae* C6706 | *∆VC1807::ptac-sfGFP* |  | This study |
| SSW11 | *E. coli* MG1655 | *araBAD::cat* | pUC18 | Crisan et al. Sci Rep. 2021 |

**Plasmids**

| Plasmid | Features | Antibiotic Resistance | Reference or Source |
| --- | --- | --- | --- |
| pET22b(+) |  | Ampicillin | Novagen (EMD Millipore) |
| pET22b(+)-*tpeV* | *PelB-tpeV-6xHis* | Ampicillin | This study |
| pEVS143 |  | Kanamycin | Dunn et al. Appl. Env. Micro. 2006. |
| pEVS143-*Aux4* | *ptac-Aux4* | Kanamycin | This study |
| pEVS143-*tpiV* | *ptac-tpiV* | Kanamycin | This study |
| pUC18 |  | Ampicillin | Norrander et al. Gene. 1983. |
